# A novel autism-associated UBLCP1 mutation impacts proteasome regulation/activity

**DOI:** 10.1038/s41398-023-02702-0

**Published:** 2023-12-21

**Authors:** Jihane Soueid, Zeinab Hamze, Joe Bedran, Maria Chahrour, Rose-Mary Boustany

**Affiliations:** 1 Department of Biochemistry and Molecular Genetics, American university of Beirut, Beirut, Lebanon; 2 Eugene McDermott Center for Human Growth and Development, University of Texas Southwestern Medical Center, Dallas, TX 75390 USA; 3 Department of Neuroscience, University of Texas Southwestern Medical Center, Dallas, TX 75390 USA; 4 Department of Psychiatry, University of Texas Southwestern Medical Center, Dallas, TX 75390 USA; 5 Center for the Genetics of Host Defense, University of Texas Southwestern Medical Center, Dallas, TX 75390 USA; 6 Peter O’Donnell Jr. Brain Institute, University of Texas Southwestern Medical Center, Dallas, TX 75390 USA; 7 Departments of Pediatrics and Adolescent Medicine, American University of Beirut Medical Center Special Kids Clinic, Neurogenetics Program and Division of Pediatric Neurology, Beirut, Lebanon

**Keywords:** Molecular neuroscience, Clinical genetics

## Abstract

The landscape of autism spectrum disorder (ASD) in Lebanon is unique because of high rates of consanguinity, shared ancestry, and increased remote consanguinity. ASD prevalence in Lebanon is 1 in 68 with a male-to-female ratio of 2:1. This study aims to investigate the impact of an inherited deletion in *UBLCP1* (Ubiquitin-Like Domain-Containing CTD Phosphatase 1) on the ubiquitin-proteasome system (UPS) and proteolysis. Whole exome sequencing in a Lebanese family with ASD without pathogenic copy number variations (CNVs) uncovered a deletion in *UBLCP1*. Functional evaluation of the identified variant is described in fibroblasts from the affected. The deletion in *UBLCP1* exon 10 (g.158,710,261CAAAG > C) generates a premature stop codon interrupting the phosphatase domain and is predicted as pathogenic. It is absent from databases of normal variation worldwide and in Lebanon. Wild-type UBLCP1 is widely expressed in mouse brains. The mutation results in decreased UBLCP1 protein expression in patient-derived fibroblasts from the autistic patient compared to controls. The truncated UBLCP1 protein results in increased proteasome activity decreased ubiquitinated protein levels, and downregulation in expression of other proteasome subunits in samples from the affected compared to controls. Inhibition of the proteasome by using MG132 in proband cells reverses alterations in gene expression due to the restoration of protein levels of the common transcription factor, NRF1. Finally, treatment with gentamicin, which promotes premature termination codon read-through, restores UBLCP1 expression and function. Discovery of an ASD-linked mutation in *UBLCP1* leading to overactivation of cell proteolysis is reported. This, in turn, leads to dysregulation of proteasome subunit transcript levels as a compensatory response.

## Introduction

Autism spectrum disorder (ASD) is a neurodevelopmental disorder diagnosed with: (1) deficits in social communication and interaction and (2) restricted, repetitive patterns of behavior [1]. ASD has strong genetic components [2, 3]. The concordance rate is 60–70% in monozygotic twins and 5–30% in siblings [4], with recurrence rates of 18% in infant siblings and 33% in multiplex families [4, 5]. ASD is diagnosed at a rate of 1 in 44 children in the USA, with males three to fourfold [6] more likely to be diagnosed than females. In Lebanon, an incidence of 1 in 68 is reported, with a male-to-female ratio of 2:1 [7, 8]. Next-generation sequencing has led to the identification of hundreds of genes linked to ASD [9–11]. The Lebanese population is genetically distinguished by a distinctive blend of DNA recombination events occurring over millennia [12, 13]. Significant shared ancestry and high rates of consanguinity make it ideally suited for genetic analysis of inherited causes of autism [14].

In this study, a novel homozygous pathogenic variant in *UBLCP1* in a Lebanese consanguineous family with ASD is described. The ubiquitin-like domain-containing C-terminal domain phosphatase 1 (UBLCP1) is a phosphatase that regulates proteasome activity. The ubiquitin-proteasome system (UPS) is responsible for the degradation of thousands of short-lived and damaged proteins. Increasing focus on the UPS role in neurodevelopmental disorders, specifically ASD, is emerging. Mutations in several genes encoding members of the UPS are associated with ASD. These genes encode ubiquitin E3 ligases (*UBE3A/B/C*, *CUL3*, *PRKN*, *FBXO40*, and *HUWE1* [15–18]) and proteasomal subunits (*PSMD10* [19] and *PSMD12* [20]).

The 26 S proteasome consists of a 20 S core particle (CP) and a 19 S regulatory particle (RP) [21]. CP is formed of subunits with peptidase activities. AAA^+^-type ATPases (Rpt1-6) together with non-ATPase subunits Rpn1, 2, and 13 form the “base” of RP, which directly binds to the CP. The “lid” of RP is composed of Rpn proteins (Rpn3, 5-12, and 15). Polyubiquitinated protein substrates are captured by RP and ubiquitin chains are cleaved off by deubiquitinases [22]. Unfolded substrates are then translocated into the CP catalytic chamber for degradation.

To maintain cell viability, UPS activity must be regulated via translational and post-translational mechanisms. Phosphatases reversibly control phosphorylation of proteasome subunits by kinases [23, 24]. UBLCP1 was the first proteasome phosphatase identified [25]. It harbors the N-terminal ubiquitin-like (UBL) domain [26] and the CTD phosphatase domain at the C-terminus. UBLCP1 interacts directly with the 19 S subunit Rpn1 through the UBL domain and dephosphorylates Rpn1-Ser361, a phosphosite required for proper assembly and activity of the 26 S proteasome [25, 27, 28]. UBLCP1 also dephosphorylates Rpt1 at multiple phosphorylation sites impairing ATPase activity [29]. Overall, these multiple dephosphorylations result in inhibition of proteasome activity by interfering with proteasome assembly [25, 29, 30].

A novel *UBLCP1* mutation is identified in a Lebanese family with ASD, resulting in a premature stop codon that disrupts UBLCP1 protein phosphatase domain, disturbing proteasome regulation and activity.

## Material and methods

### Subjects and specimens

Blood was collected from a 20-year-old ASD patient, who fulfilled DSM-5 criteria for autism, his parents, and two healthy siblings according to a protocol and consent forms reviewed and approved by the Institutional Review Board (IRB) of the American University of Beirut (IRB protocol number: BIOCH.RB.06, protocol name: Autism susceptibility genes in the Lebanese population). All experimental protocols were carried out in accordance with institutional guidelines. Informed consent was obtained by CITI-certified investigators from all family members, including both parents, siblings, and by parents on behalf of the affected individual. Blood samples from a cohort of 100 unaffected controls were collected. DNA samples were extracted from peripheral blood using QIAamp® blood midi kit (Qiagen, Inc., Valencia, CA).

### Whole exome sequencing, data processing, and variant identification

Whole exome sequencing (WES) was performed on all family members, including the individual with ASD, both parents and healthy siblings. DNA was sequenced on the Illumina HiSeq 2000 platform at the Broad Institute (Massachusetts Institute of Technology) with a mean target coverage of 90% at ≥20× and a mean read depth of 102×. WES data was processed following best practices recommended by the Broad Institute [31]. Reads were aligned to the human reference genome version GRCh37/hg19 using the Burrows-Wheeler Aligner (BWA, version 0.7.10). Duplicate reads were removed using Picard (version 1.117). Local realignment and quality recalibration were performed using the Genome Analysis Toolkit (GATK; version 3.3). Variants (single nucleotide variants (SNVs) and insertions or deletions (indels) were detected using GATK with HaplotypeCaller. Quality control checks for (i) duplicate samples, (ii) samples per platform, (iii) genome call rate, (iv) missingness rate, (v) singleton rate, (vi) heterozygosity rate, (vii) homozygosity rate, (viii) Ti/Tv ratio, (ix) inbreeding coefficient, and (x) sex inference were performed. Variant call format (VCF) files for SNVs and indels were annotated with ANNOVAR using allele frequencies from the 1000 Genomes project (2015; 1000 G), the Genome Aggregation Database (gnomAD), and the Greater Middle East Variome Project (GME). Annotated VCF files were uploaded into an SQL database for working storage and analysis. Genome data was stored and analyses were performed on the Texas Advanced Computing Center (TACC) high-performance computing server, a resource of the University of Texas (Austin, TX). Sanger sequencing was performed on DNA samples from the affected individual and family members to validate the WES finding of the *UBLCP1* variant (g. 158,710,261CAAAG > C) by amplification using primers UBLCP1_Foward 5′-GCTGGTGTTGTCAGGTTCAG-3′ and UBLCP1_Reverse 5′-GGGGTCACTGATGAAAAATGA-3′).

### Dermal fibroblast derivation and expansion

Skin biopsy from the proband and from control age-matched Lebanese males were transferred into sterile glass Petri dishes containing 4 ml freshly prepared fibroblast media (Dulbecco’s modified Eagle’s medium high glucose with GlutaMAX (Life Technologies, Carlsbad, CA, USA), 10% certified fetal bovine serum (Life Technologies, Carlsbad, CA, USA), 1% antibiotics (Sigma, St. Louis, MO, USA)). Four scratches were made in each well of a 12-well plate using a 19-gauge needle to produce a checkerboard pattern. Biopsy was minced into small 2-mm pieces by sharply cutting edges using a new disposable stainless-steel blade scalpel. The biopsy pieces were transferred onto the 12-well culture plate (3 pieces/well) to the intersection of two scratches so that cells could adhere and migrate from the sample onto the culture plate. Tissue pieces were allowed to dry for 3 minutes (min) under the hood, and a few drops of media were added. After incubating for 30 min at 37 °C, an appropriate volume of media was added to submerge the tissue pieces. Explants were subjected weekly to a complete medium change. As fibroblasts grew out from the explants and expanded to 50% confluency, tissue pieces were removed. Fibroblast cultures were established from skin biopsies after 3–4 weeks. Fibroblasts were detached from the flask using prewarmed 37 °C Trypsin (Lonza, Houston, TX, USA) and split into larger culture flasks for expansion. Cells were frozen at a density of 1.0 × 10^6^ cells/milliliter/cryovial. All cell lines used/frozen tested negative for mycoplasma contamination.

### Fibroblast read-through experiments and proteasome inhibition

Early passage primary skin fibroblasts were plated in 25 cm^2^ flasks and grown to 80% confluency. For read-through experiments, cells were treated with gentamicin (Sigma-Aldrich, St. Louis, MO, USA) at 100 μM to promote premature termination codon (PTC) read-through, then harvested after 24 hours (hr) of treatment. For proteasome inhibition, cells were treated with MG132 (Calbiochem, San Diego, CA, USA) at 10 μM, or just with DMSO vehicle, then harvested after 10 hr of treatment. Control fibroblasts without treatment, control fibroblasts treated with gentamicin or MG132, patient-derived fibroblasts without treatment, and patient-derived fibroblasts treated with gentamicin or MG132 were assayed using biological triplicates, and technical triplicates for each biological replicate.

### Proteasome activity

Protein samples (2 μg) were incubated with 100 μM succinyl-Leu-Leu-Val-Tyr-7-amido-4-methylcoumarin (Suc-LLVY-AMC) in a proteasome activity buffer (50 mM Tris–HCl (pH 7.5), 40 mM KCl, 5 mM MgCl_2_, 1 mM DTT, 2 mM ATP) for a final volume of 100 μl. The release of fluorescent free-AMC was quantified at an excitation of 380 nm and emission of 460 nm. After 1 hr incubation in the dark at 37 °C, fluorescence was measured using a spectrophotometer. All experiments were done using biological triplicates, as well as technical triplicates for each biological replicate.

### Western blot and immunohistochemistry

For western blot analysis, experiments were performed using biological triplicates, as well as technical triplicates for each biological replicate. Cells were harvested and lysed in RIPA lysis buffer with a protease inhibitor cocktail (Roche, Basel, Switzerland). Protein concentrations of whole cell lysates were determined by Bradford protein assay using Coomassie Plus Protein Assay Reagent (Fisher Scientific, Ottawa, ON, Canada). Proteins were separated after denaturation for 5 min at 95 °C on a 10% acrylamide gel in a Tris-glycine running buffer system and transferred onto a PVDF membrane with a Tris-glycine buffer system using a wet transfer unit (BioRad, Hercules, CA, USA) at 100 volts for 40 min. Membranes were blocked using 5% bovine serum albumin and incubated with antibody in TBST overnight at 4 °C with antibodies to Ubiquitin (1:1000; Cell Signaling, Massachusetts, USA) and GAPDH (1:1000; Cell Signaling, Massachusetts, USA) or Vinculin (1:1000; Cell Signaling, Massachusetts, USA). Membranes were then incubated in HRP-conjugated secondary antibodies. Signals were detected by chemiluminescence using the SuperSignal West Femto Maximum Sensitivity Substrate (ThermoFisher Scientific, MA, USA) and visualized using ChemiDoc MP Imaging System (BioRad, Hercules, CA, USA). Quantification was performed using ImageJ. Bands corresponding to the protein of interest, or their respective loading control (GAPDH or Vinculin), were selected, as well as an additional “blank” background band which was used to subtract background signal/noise from the measurements. The resulting values were then normalized to their respective reference band (GAPDH or Vinculin).

For immunofluorescence experiments, cells previously plated on coverslips in 24-well plates were fixed with 4% paraformaldehyde/PBS for 20 min, washed three times with PBS, and incubated for 45 min in blocking buffer (PBS, 10% fetal calf serum, and 0.1% Triton X-100). Cells were incubated overnight with primary antibody (anti-UBLCP1, 1:200, Sigma, St. Louis, MO, USA), then secondary antibody coupled with fluorescence. The nucleus was counterstained with Hoechst dye. Fluorescence was visualized on an inverted confocal microscope (Leica, Germany). Parameters (such as brightness, contrast, and fluorescence threshold) remained unchanged and were standardized for all quantified images. When comparing mutated versus control fibroblasts, parameters were set for low-brightness signals within the dynamic range, and maintained equal for all images. Quantification of fluorescence intensity was performed using the ImageJ software.

For brain tissue collection and processing, C57BL-6 adult mice (*n* = 3, 2 males and 1 female) were perfused using 4% paraformaldehyde (4% PFA), and brains harvested. Post-natal day 4 C57BL-6 mice (*n* = 4, 2 males, and 2 females) were sacrificed by decapitation, dissected, and fixed in 4% PFA for 4 hours. Fixed brains were subsequently immersed in 20% sucrose for 24 hours before freezing, then embedded in a cryo-embedding matrix and frozen. Frozen brains were sectioned using a cryotome at a thickness of 20 microns per section. Antibody concentrations used for brain tissue immunofluorescence are as follows: anti-Map2, 1:500 (Invitrogen, MA, USA); anti-UBLCP1, 1:200 (Sigma, St. Louis, MO, USA); anti-CD11b, 1:50 (Cell signaling, Massachusetts, USA); anti-TH, 1:500 (Chemicon, Massachusetts, USA); anti-Olig2, 1:100 (Millipore, Massachusetts, USA). Fluorescence was visualized on an inverted confocal microscope (Leica, Germany). No randomization or blinding was used for these mice. All animal experiments were performed with approval from the Animal Care Committee of the American University of Beirut, Lebanon.

### RNA extraction and qRT-PCR

Total RNA was extracted from fibroblasts and reverse transcribed using RevertAid Reverse Transcriptase (ThermoFisher Scientific, MA, USA) with 100-1000 ng of input RNA and random primers (ThermoFisher Scientific, MA, USA). Quantitative real-time PCR reactions (qRT-PCR) were performed in 96- or 384-well plates using specific primers (TIB MOLBIOL, Germany), and the iQTM SYBR® Green Supermix (BioRad, CA, USA) was used as a fluorescent detection dye in a final volume of 10 μl (CFX96TM Real-Time PCR (BioRad, CA, USA)). Each reaction was performed using biological triplicates, as well as technical triplicates for each biological replicate. All results are normalized to β-actin or GAPDH mRNA levels and calculated using the 2^∆Ct^ method. Melt curve analysis was applied to characterize generated amplicons and to control contamination by unspecific by-products.

### Statistical analysis

Statistical analysis was conducted using GraphPad Prism 6 software (GraphPad software, CA, USA). Continuous data was expressed as mean ± SEM. Statistical analysis was performed using two-tailed Student’s *t* test and one-way analysis of variance (ANOVA) followed by Tukey post hoc test for multiple group comparisons. Values of *P* < 0.05 were considered statistically significant.

## Results

### Clinical presentation, whole exome sequencing, and variant identification

**Fig. 1:**
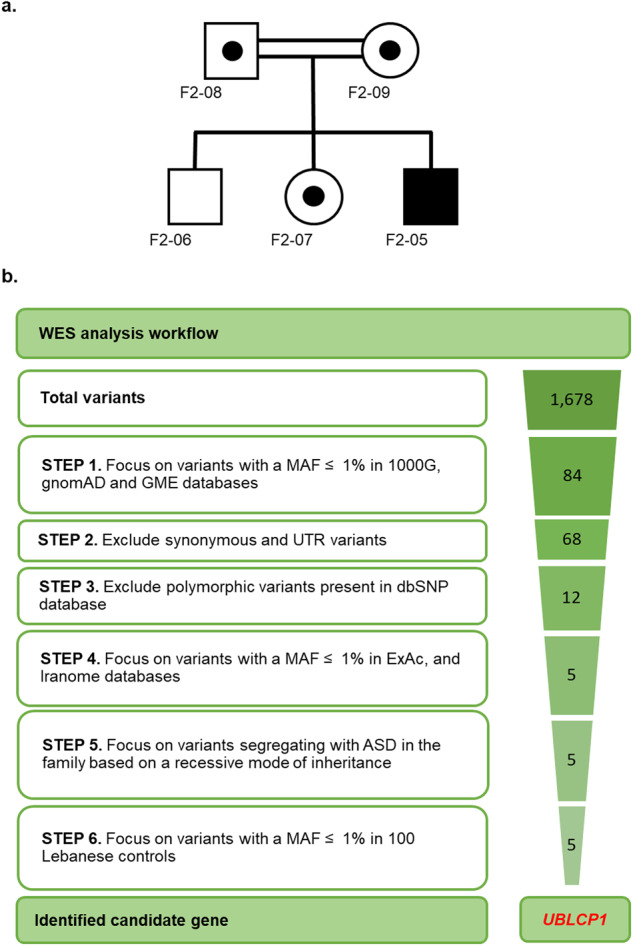
Whole-exome sequencing data analysis strategy. **a** F2 Family pedigree and segregation of the *UBLCP1* variant g.158,710,261CAAAG > C: open circles and squares represent unaffected females and males, respectively; closed square represents affected male homozygous for the variant; half-filled circles and squares denote unaffected heterozygous carriers. **b** WES data analysis and filtering strategy used to prioritize identified variants in family F2. The workflow diagram illustrates the number of variants identified at each step (gradient) of the filtering process, assuming a homozygous recessive model of inheritance. A brief description of each step of the filtering process (steps 1–6) is shown at the left of the diagram. Briefly, after alignment and post-alignment processing and the removal of variants with missing genotype data or multiple alleles, a total number of variants was identified across sequenced exomes. Step 1 involved mining for variants with a minor allele frequency (MAF) ≤ 1% using data from the 1000 Genomes project (1000 G), Genome Aggregation Database (gnomAD), and Greater Middle East Variome Project (GME). Step 2 involved excluding all synonymous and UTR variants. Step 3 excluded polymorphic variants found in dbSNP. Step 4 involved selecting variants with global mean allele frequency ≤1% in ExAc and Iranome databases. Step 5 involved confirming by Sanger sequencing the genotypes in all family members, and keeping variants with proper segregation within the family. Step 6 relied on genotyping of candidate variants in a local database consisting of 100 Lebanese controls and keeping variants with an MAF ≤ 1%. Steps 1–6 led to prioritization of the variant in the candidate gene *UBLCP1*.

**Fig. 2:**
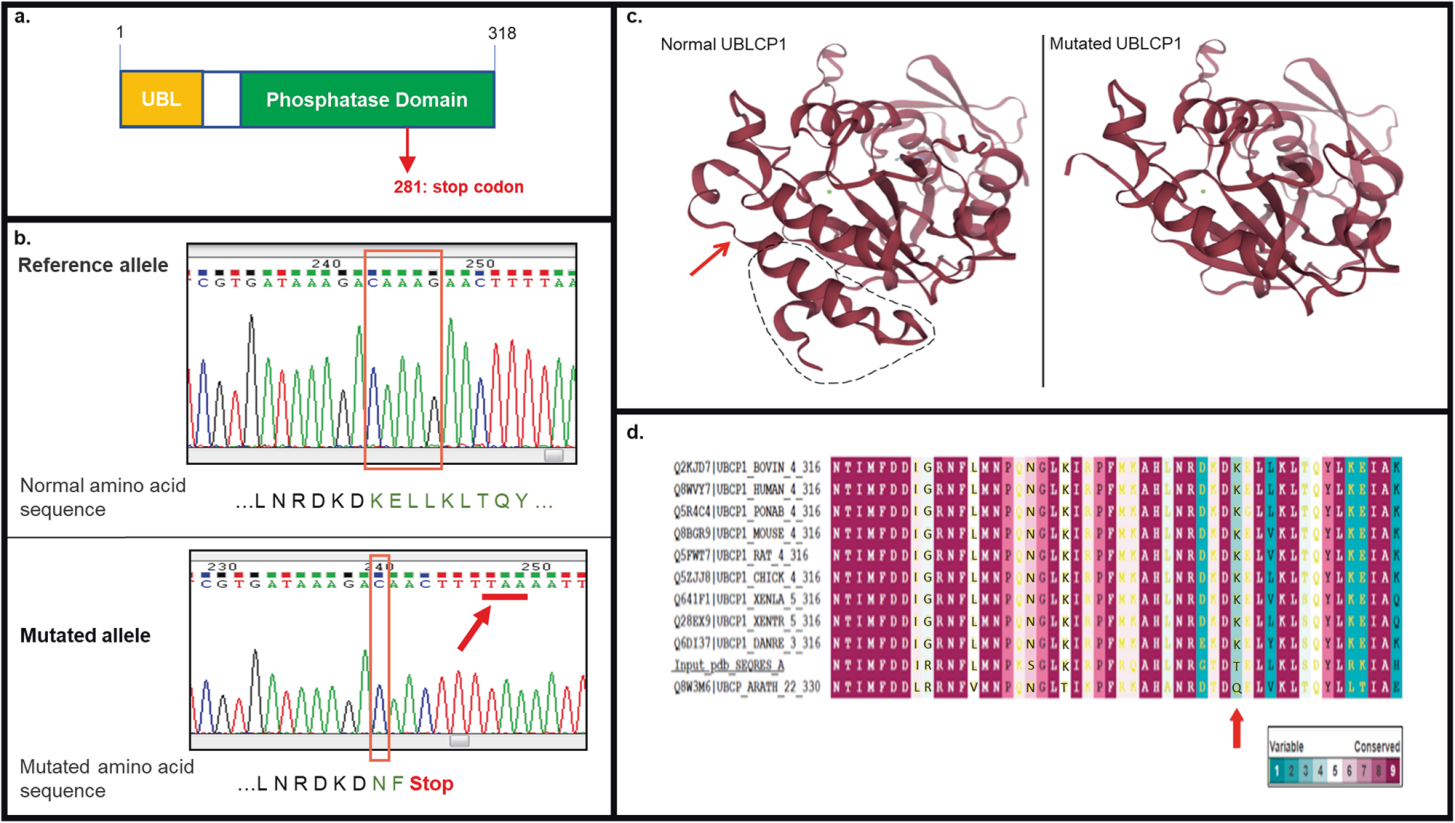
*UBLCP1* mutation analysis. **a** Schematic representation of the UBLCP1 protein domains with a predicted stop codon (red arrow) within the phosphatase domain (green). UBLCP1 ubiquitin-like domain or UBL (yellow) binds 26 S of the UPS. **b** Sanger sequencing of *UBLCP1*: del g.158,710,261CAAAG > C results in a premature stop codon TAA (red arrow) (p.K282Nfs*3). **c** Three-dimensional protein modeling of UBLCP1 using the ConSurf server [57], with the predicted structural effect of the identified ASD variant depicting the p.K282Nfs*3 mutation as leading to protein truncation at the C-terminus (dashed line). **d** Evolutionary conservation of amino acids around the position affected by the mutation identified in UBLCP1 (red arrow) in a Lebanese family with ASD (blue indicates high conservation; red indicates low conservation).

F2-05, a boy diagnosed with ASD, is the third child of a Lebanese family from a first-cousin marriage (Fig. 1a). Previous microarray analysis in the affected individual showed the absence of known or predicted ASD-associated CNVs. To identify putative causative variants, whole exome sequencing (WES) was performed on all family members, including the proband, both parents, and both unaffected siblings. Assuming a homozygous recessive model of inheritance, led to the identification of variants in 5 candidate genes (Fig. 1b, Table 1). Of these variants, three were intronic identified in candidate genes *GABRB2*, *HDAC9,* and *TAS1R2*. These variants were all predicted to be probably benign by MutationTaster. Additional transcriptional analysis of *GABRB2*, which encodes the γ-aminobutyric acid (GABA) A receptor beta 2 subunit, ruled out splice site involvement and showed no functional impact for the identified variant (data not shown). The remaining 2 identified variants were non-synonymous exonic: a missense variant in *WNK1* (g.1,005,622 C > T, p.P2488L) and a frameshift variant in *UBLCP1* (g.158,710,261CAAAG > C, p.K282Nfs*3). The *WNK1* variant was predicted to be disease causing by MutationTaster, PolyPhen2, and SIFT, but was predicted as a polymorphism by UMD predictor. In addition, the mutated residue was not conserved across species (Table 1). This homozygous frameshift deletion in *UBLCP1*, predicted to be deleterious by MutationTaster (Table 1), leads to a downstream premature termination codon (PTC) resulting in truncation of the phosphatase domain (Fig. 2a–c). The affected residue was partially conserved across species (Fig. 2d). To confirm WES result, we performed Sanger sequencing on samples from all family members. The homozygous g.158,710,261CAAAG > C variant was confirmed in the proband. In addition, it was confirmed that both parents and an unaffected sister are heterozygous carriers, and the unaffected brother is homozygous for the reference allele. The variant was absent from 100 unaffected and unrelated Lebanese subjects, and it has not been reported in the medical literature or in disease-related variant databases (Human Gene Mutation Database (HGMD) or ClinVar).

**Table 1:** Prioritized candidate variants in family F2.

Variant position (hg19)	Gene	Type of variation	Pathogenicity predictions				Expression in the brain	Conservation of affected residue among species^a^	Conclusion
			Mutation Taster	Polyphen2	SIFT	UMD Predictor: score; prediction			
chr5:160,761,721 C > A	*GABRB2*	Intronic	Probably harmless	N.A.	N.A.	N.A.	High	N.A.	Prioritized: *UBLCP1* Excluded: *GABRB2*^b^, *HDAC9*, and *TAS1R2*
chr7:18,788,574 G > A	*HDAC9*	Intronic	Probably harmless	N.A.	N.A.	N.A.	Medium	N.A.	
chr1:19,185,981 C > T	*TAS1R2*	Intronic	Probably harmless	N.A.	N.A.	N.A.	Not detected	N.A.	
chr12:1,005,622 C > T	*WNK1*	Exonic (P2488L)	Disease causing	Probably damaging	Not tolerated	36; Polymorph.	High	Not conserved	
chr5:158,710,261CAAAG > C	*UBLCP1*	Exonic (K282Nfs^a^3)	Disease causing	N.A.	N.A.	N.A.	High	Partially conserved	

^a^Conservation amino-acid sequences of chimp, rhesus macaque, mouse, cat, chicken, claw frog, pufferfish, zebrafish, fruitfly, and worm are aligned with corresponding human sequence of mutated genes.

^b^GABRB2 was excluded based on transcriptional analysis that ruled out splice site involvement. *NA* not assigned.

### Characterization of wild-type UBLCP1 expression and localization in the brain

**Fig. 3:**
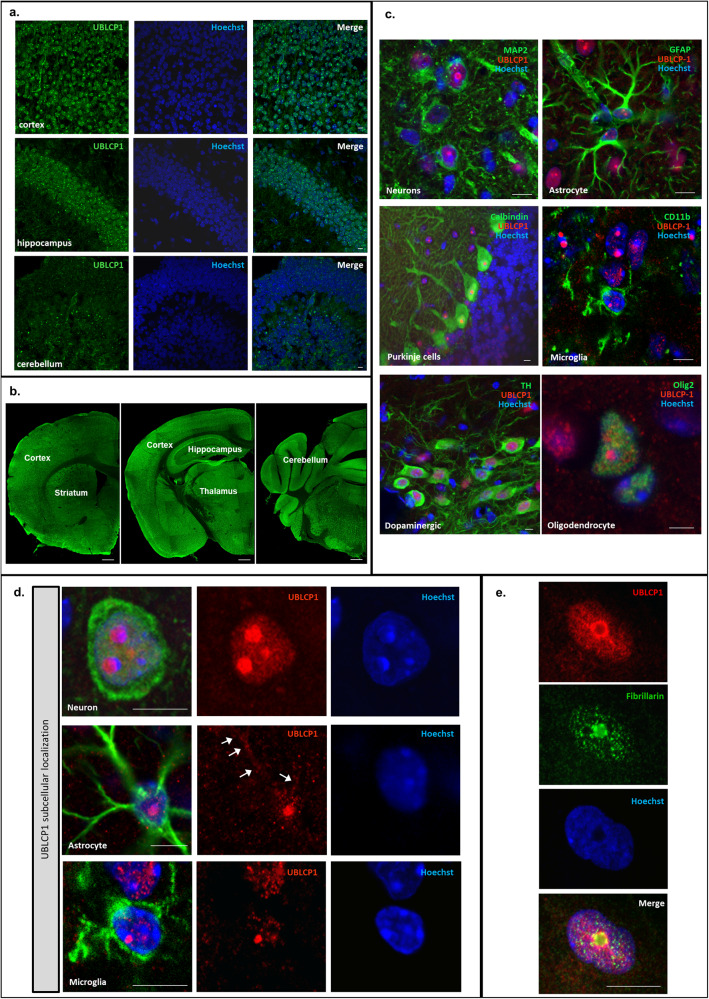
UBLCP1 protein expression pattern in mouse brain and subcellular localization in different cell types in the brain. **a** UBLCP1 is expressed in wild-type post-natal day 4 mouse brain. Sample images from the cortex, hippocampus, and cerebellum are shown. Brain sections were immunolabeled for UBLCP1 (green). Nuclear DNA was labeled with Hoechst (blue). **b** Representative images of different coronal brain sections of a wild-type adult mouse (28 weeks old) with multiple regions shown. UBLCP1 is expressed in the cortex, striatum, thalamus, hippocampus, and cerebellum. **c** UBLCP1 localization in neurons is depicted in the left panels with representative images from neurons in the diencephalon, Purkinje cells in the cerebellum, and dopaminergic neurons in the ventral tegmental area. Brain sections were immunolabeled for UBLCP1 (red), and neuronal markers MAP2 (green, top panel), calbindin (green, middle panel), or TH (green, lower panel). UBLCP1 expression in the glial population is depicted in the panels on the right. UBLCP1^+^ cells (red) are co-labeled with markers of astrocytes (GFAP, green, top panel), microglia (CD11b, green, middle panel), and oligodendrocytes (Olig2, green, lower panel). Nuclear DNA was labeled with Hoechst (blue in all). **d** UBLCP1 is identified in the nucleolus of neurons, astrocytes, and microglia, and also in the nucleoplasm in neurons. **e** Nuclear UBLCP1 (in red) co-localizes with the nucleolar marker fibrillarin (in green). All nuclei in the figure were counterstained blue with Hoechst. **b** Scale bar: 500 µm. **a**, **c**–**e** Scale bar: 10 µm.

To assess the relevance of UBLCP1 to ASD, its expression in the mammalian brain was examined. UBLCP1 expression was detected in early post-natal brain (post-natal day 4) (Fig. 3a) and in adult mouse brain (Fig. 3b) by immunohistochemical staining. UBLCP1 was present in all regions of the brain including cortex, diencephalon, rhombencephalon, hippocampus, cerebellum, and ventral tegmental area. This pattern of expression is consistent with the distribution of *Ublcp1* mRNA as determined by in situ hybridization data from the Allen Brain Atlas project [32]. There was no difference noted between post-natal day 4 and adult mice without the impact of male and female gender on mouse brains at the two ages.

Double immunolabeling was used to assess UBLCP1 cellular and subcellular localization in the adult. The presence of UBLCP1 was observed in neurons (NeuN and MAP2 positive), dopaminergic neurons [tyrosine hydroxylase (TH) positive], and Purkinje cells (calbindin positive). UBLCP1 was also expressed in the glial population, in astrocytes (GFAP positive), microglia (CD11b positive), and oligodendrocytes (Olig2 positive) (Fig. 3c). Interestingly, UBLCP1 expression in glial cells is mostly localized to the nucleolus. Neuronal cells display additional expression of UBLCP1 in the nucleoplasm (Fig. 3d). UBLCP1 colocalized with the nucleolar protein fibrillarin in neurons, a component of the small nuclear ribonucleoprotein complex required for processing of pre-rRNA molecules [33] (Fig. 3e). A weak signal was detected in the cytoplasm of some but not all cells on post-natal day 4 and in adult mice.

### The *UBLCP1* ASD mutation (del g.158710261CAAAG > C) alters UBLCP1 levels in fibroblasts

**Fig. 4:**
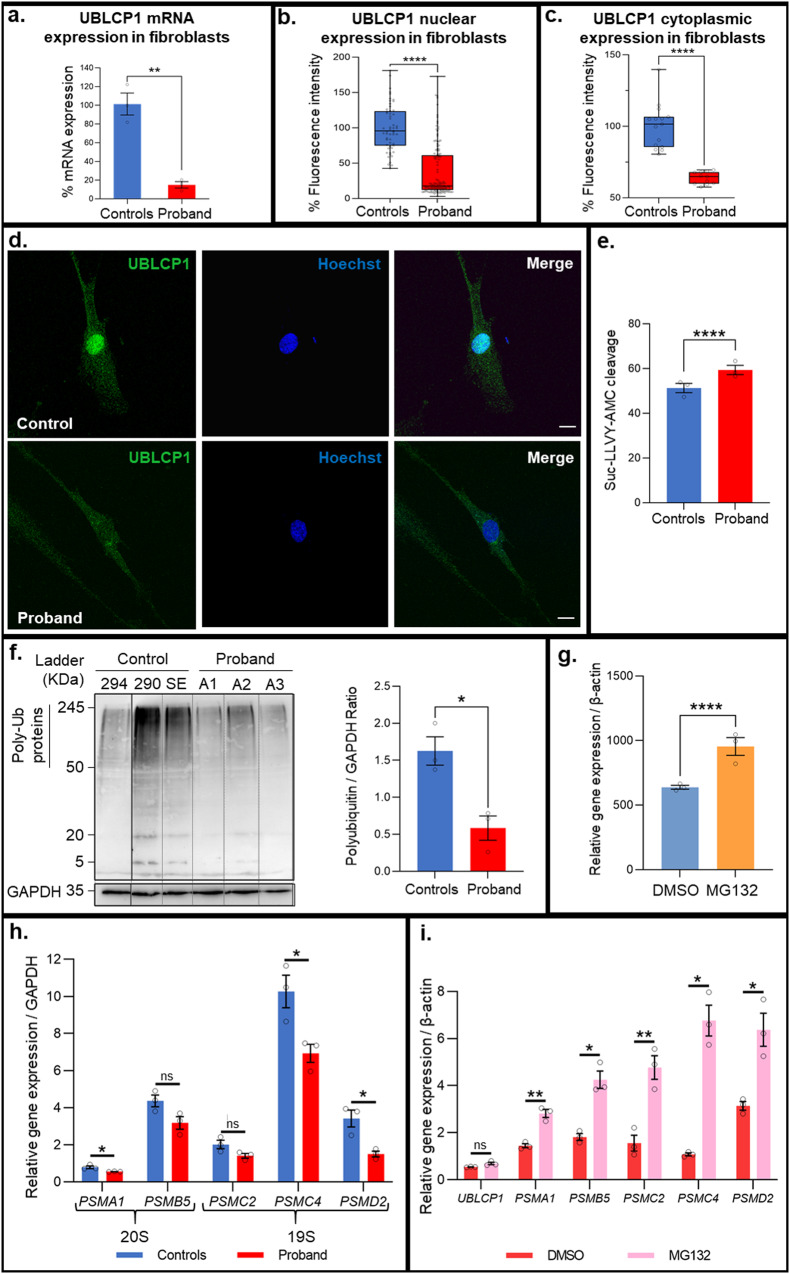
UBLCP1 ASD mutation results in decreased expression, enhanced proteasomal degradation of ubiquitinated proteins, and altered differential RNA expression of proteasome subunits. **a** *UBLCP1* mRNA levels in fibroblasts from ASD patient compared to unaffected controls. **b** UBLCP1 nuclear protein expression in fibroblasts from ASD patient (*n* = 89) compared to unaffected controls (*n* = 90). **c** UBLCP1 cytoplasmic protein expression in fibroblasts from ASD patient (*n* = 107) compared to unaffected controls (*n* = 105). **d** Immunostaining of unaffected control fibroblasts (upper images) and fibroblasts from the ASD patient (lower images). UBLCP1 expression is in green, and the nucleus is counterstained with Hoechst in blue. ****P < 0.0001; Student’s *t* test; mean ± SEM. Scale bar: 10 µm. **e** Enhanced proteasome activity in fibroblasts from the proband compared to unaffected controls. Fibroblasts used in these experiments were at the same passage number. Proteasome activity was measured using the fluorogenic substrate Suc-LLVY-AMC. Student’s *t* test, *P = 0.0498, *n* = 3 in each group. **f** Decreased polyubiquitination detected by western blot in fibroblasts from the proband compared to unaffected controls. Ubiquitin and GAPDH were detected by western blot (left panel). Equivalent amounts of total protein were loaded into each lane within the same gel. Quantification is shown on the right. Relative levels of polyubiquitinated proteins (area of interest included 50–250 kDa) to GAPDH were quantified with *n* = 3 per group. *P = 0.0145; Student’s *t* test; mean ± SEM. **g** Increased *UBLCP1* mRNA levels in fibroblasts from three independent controls treated with 1uM MG132 for 10 h compared to the same controls treated with DMSO vehicle only. **h** A general tendency for reduced mRNA expression of proteasome subunits (*PSMA1*, *PSMB5*, *PSMC2*, *PSMC4*, and *PSMD2*) from the proband’s cells compared to expression in cells derived from unaffected controls was observed. *n* = 3 per group. *P < 0.05; Student’s *t* test; mean ± SEM. **i** Enhanced expression of *UBLCP1* and proteasome subunits (*PSMA1*, *PSMB5*, *PSMC2*, *PSMC4*, and *PSMD2*) mRNA extracted from proband cells treated with 1uM MG132 for 10 h compared to expression in proband cells treated only with DMSO vehicle. *n* = 3 per group. *P < 0.05; **P < 0.01; Student’s *t* test; mean ± SEM.

The described UBLCP1 frameshift mutation leads to a premature stop codon. The impact on mRNA levels was assessed by qRT-PCR. Analysis revealed significantly reduced *UBLCP1* mRNA levels in fibroblasts from the affected individual compared to unaffected controls (Fig. 4a). Immunostaining of fibroblasts showed that UBLCP1 localized mostly to the nucleus in control fibroblasts and exhibited a diffuse distribution in the cytoplasm (Fig. 4d, upper panel), whereas mutant UBLCP1 had a marked reduction in expression in fibroblasts from the ASD patient (Fig. 4d, lower panel). Quantification showed an 80% and 40% decrease in fluorescence of mutant protein within the nucleus and cytoplasm, respectively, in ASD patient fibroblasts (Fig. 4b, c).

### Proteasome activity is enhanced in fibroblasts with the *UBLCP1* (del g.158710261CAAAG > C) mutation

The ASD mutation identified in family F2 is a deletion within exon 10 of *UBLCP1* (g. 158,710,261CAAAG > C) and is predicted to generate a stop codon that interrupts the phosphatase domain of the protein. To study the effect of this mutation on the UPS, proteasome activity was measured in fibroblasts using a fluorogenic synthetic peptide substrate, Suc-LLVY-AMC, which is converted to a fluorescent degradation product, 7-amino-4-methylcoumarin (AMC) upon cleavage by the proteasome. Fibroblasts from the proband displayed a statistically significant increase in the rate of Suc-LLVY-AMC cleavage compared to fibroblasts from unaffected controls (Fig. 4e). In addition, Western blot analysis uncovered a statistically significant decrease in the level of ubiquitinated proteins in fibroblasts from the proband compared to those from unaffected controls (Fig. 4f).

### Differential expression of 26 S proteasome subunits

In addition to proteolysis in cells, the UPS impacts the regulation of the cell cycle, signal transduction, apoptosis, and antigen presentation, as well as other cellular functions. Proteasome homeostasis is tightly regulated. Mammalian cells respond to impairment of cellular proteasomal enzyme activity through a coordinated upregulation of proteasome subunits at both the transcriptional and translational levels. The hypothesis is that defective UBLCP1 protein leads to decreased inhibition of proteasome activity. The effect of the UBLCP1 ASD mutation on RNA expression of different proteasome subunits (*PSMA1*, *PSMB5*, *PSMC2*, *PSMC4*, and *PSMD2*) in patient-derived fibroblasts was investigated by qRT-PCR. A general tendency for reduced RNA expression of all the subunits in the proband’s cells compared to expression in cells from unaffected controls was observed. This reduction was statistically significant for *PSMA1* (*P* = 0.0318), *PSMC4* (*P* = 0.0291), and *PSMD2* (*P* = 0.0165) (Fig. 4h). When proband cells were treated with MG132, a proteasome inhibitor, the mRNA levels of the 5 subunits increased significantly compared to DMSO treated cells (*PSMA1* (*P* = 0.0053), *PSMB5* (*P* = 0.0128), *PSMC2* (*P* = 0.0084), *PSMC4* (*P* = 0.0124), and *PSMD2* (*P* = 0.0369) (Fig. 4i). Although mutant *UBLCP1* mRNA levels were slightly increased, this difference did not reach statistical significance (P = 0.1096). The effect of proteasome inhibition with MG132 on wild-type *UBLCP1* gene expression was determined in control fibroblasts. *UBLCP1* mRNA levels were significantly increased in treated cells as compared to DMSO-treated cells (P = 0.0384) (Fig. 4g).

### Gentamicin treatment restored expression and function of mutant UBLCP1 in ASD patient-derived fibroblasts

**Fig. 5:**
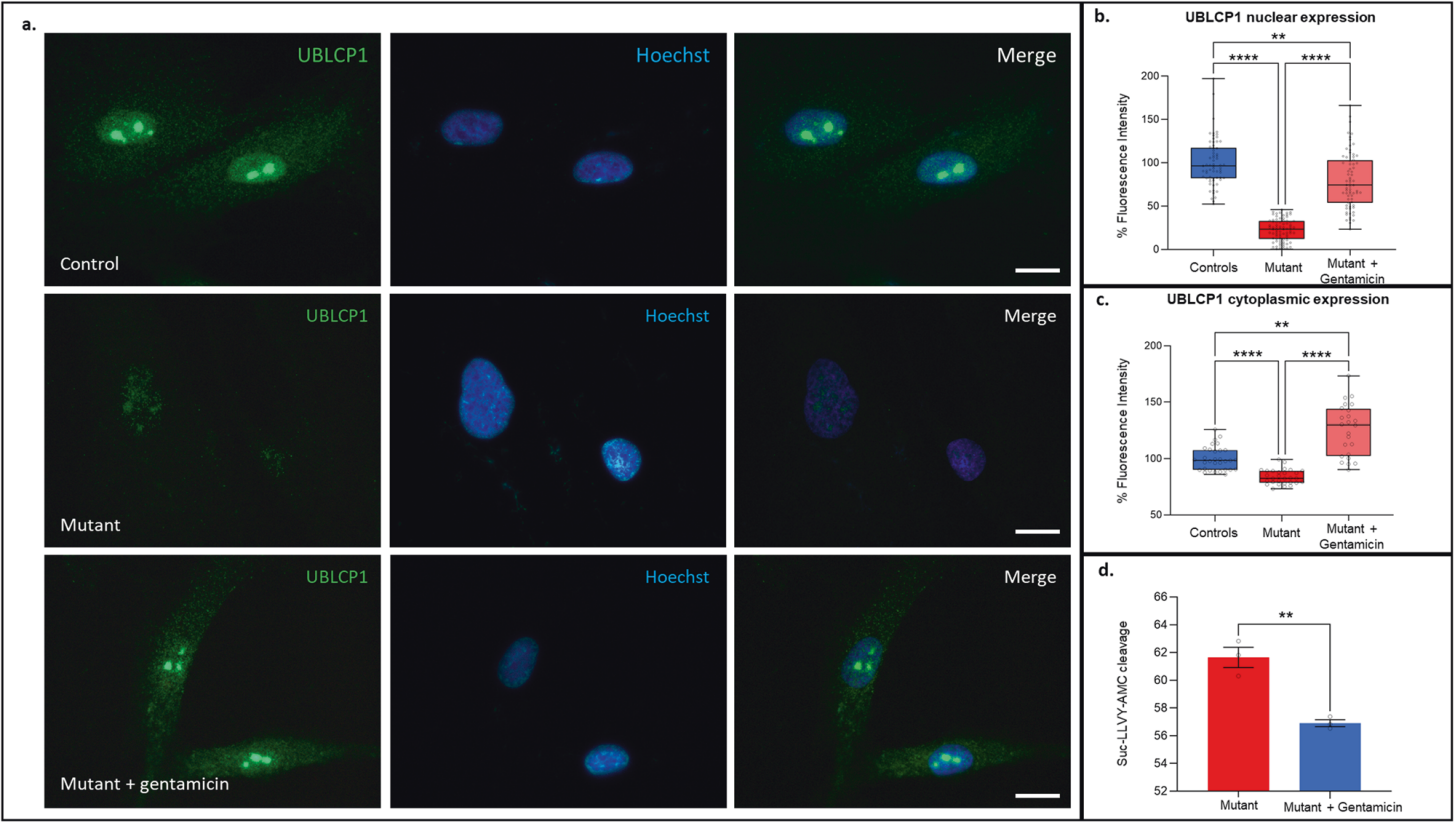
UBLCP1 mRNA expression and function are restored in fibroblasts from ASD patient after cells treatment with gentamicin. **a** Control fibroblasts (upper panel) and fibroblasts with mutant UBLCP1 (del g.158710261CAAAG > C) (middle panels) treated for 24 h with 100 µM gentamicin (lower panels), then immunostained with antibody against UBLCP1 (green) and counterstained with Hoechst (blue). **b** UBLCP1 nuclear protein expression and **c** UBLCP1 cytoplasmic protein expression in fibroblasts from unaffected controls (*n* = 66), from ASD patient (*n* = 79), and cells treated with gentamicin (*n* = 72). ***P = 0.0001, ****P < 0.0001; mean ± SEM. Scale bar: 10 µm. **d** Decrease in proteasome activity is observed in fibroblasts from ASD patient cells after treatment with 100 µM gentamicin for 24 h compared to the same fibroblasts without treatment. Proteasome activity was measured using the fluorogenic substrate Suc-LLVY-AMC. Student’s *t* test, *P = 0.0152, *n* = 3 experiments per group.

Gentamicin, an aminoglycoside antibiotic, can promote premature termination codons (PTC) read-through, restoring full-length protein without inducing significant misreading at normal stop codons [34]. Untreated fibroblasts generated from the ASD patient revealed significantly reduced UBLCP1 protein expression in the nucleus and in cytoplasm compared to control fibroblasts. Treatment of patient fibroblasts with gentamicin restored UBLCP1 expression in the nucleus and cytoplasm (Fig. 5a). Quantification revealed that UBLCP1 expression was significantly reduced in the nucleus and the cytoplasm to 22.4% ± 1.4% and 83.8% ± 1.2% of levels observed in control fibroblasts, respectively. Treatment with gentamicin upregulated UBLCP1 expression in nucleus and cytoplasm to 79.5 ± 3.8% and 125.6 ± 4.7% of levels observed in control fibroblasts, respectively (Fig. 5b, c).

To verify if UBLCP1 proteasome inhibitor function was restored, proteasome activity was investigated after treatment with gentamicin. Fibroblasts generated from the ASD patient displayed a statistically significant decrease in the rate of Suc-LLVY-AMC cleavage after treatment with gentamicin, confirming restored function (Fig. 5d).

## Discussion

In this study, whole exome sequencing of a consanguineous Lebanese family with a boy affected with ASD, led to the identification of a novel candidate gene, *UBLCP1*. The gene encodes a protein with a phosphatase domain implicated in the regulation of the proteasome. Under normal conditions, UBLCP1 functions to inhibit or downregulate proteasome activity.

Histological analysis in the mouse revealed that UBLCP1 is ubiquitously expressed early during postnatal development and in the adult brain. This consolidates the idea that UBLCP1 plays an essential role in cellular functioning. A defective UBLCP1 may cause dysregulation in cellular homeostasis leading to altered development and function of the central nervous system. Ubiquitous expression in neuronal and glial cell types cannot but have a role to play in the pathogenesis of ASD. Functional analysis of fibroblasts bearing truncated UBLCP1 revealed an increase in proteasome activity, and subsequent decrease in polyubiquitinated protein levels. This data confirms the hypothesis that mutant UBLCP1, in this instance fails to phosphorylate the proteasome, leading to decreased inhibition, hence, increased proteasomal activation. Dysfunction of the UPS is observed in many diseases, including disorders of the brain [30]. Numerous mutations in genes encoding proteins within the UPS are linked to neurodegenerative and neurodevelopmental disorders, including ASD [35, 36].

Guo et al. showed that UBLCP1 localizes to the nucleus due to its interaction with nuclear proteasomes via the Lys44 residue within the UBL domain [25]. In this study, UBLCP1 was detected primarily in the nucleus, but also at lower levels in the cytoplasm. These results are in line with observations of proteasome location in the nucleolus and the cytoplasm [37]. Despite the opinion that proteasomes degrade proteins inside the nucleus, recent studies diverge and report that catalytically active proteasomes exist almost exclusively in the cytosol [38]. Here, there is a 40% decrease in expression of mutant UBLCP1 in the nucleus. The hypothesis here is that wild-type UBLCP1, by binding and dephosphorylating the proteasome decreases its assembly, and contributes to its nuclear localization in a dissociated state. In the case of mutant UBLCP1 truncated within the phosphatase domain, it binds to the proteasome via the UBL domain but does not dephosphorylate it, hence, resulting in increased assembly and trafficking of active proteasome to the cytoplasm.

**Fig. 6:**
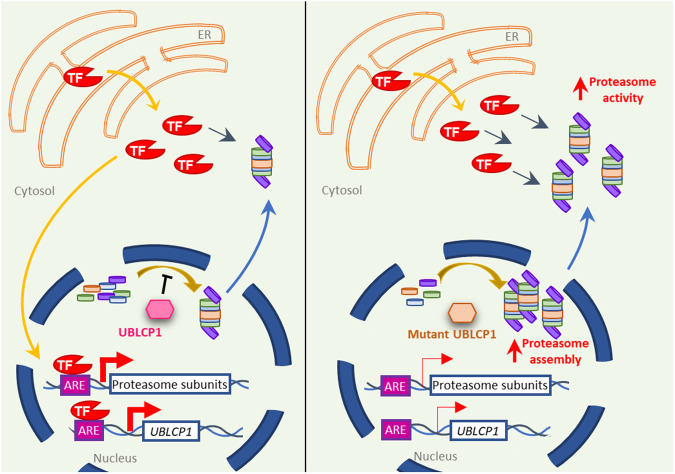
Model of the molecular pathway disrupted as a consequence of the UBLCP1 ASD mutation. (Left) The activity and regulation of the proteasome under normal conditions. A transcription factor (TF) is released from the endoplasmic reticulum (ER) to the cytosol, where it is degraded by active proteasomes. With proteotoxic stress, the TF enters the nucleus and binds to antioxidant response element (ARE) core sequences found upstream of most proteasome subunit genes, including *UBLCP1*. These levels of gene transcription maintain a pool of free subunits from which proteasome assembly can rapidly be generated. In the nucleus, wild-type UBLCP1 (pink) restrains the assembly of the proteasome. Fully assembled proteasomes are translocated to the cytosol, where they are active. (Right) The activity and regulation of the proteasome in ASD patient-derived cells with the *UBLCP1* (del g.158,710,261CAAAG > C) mutation. In the nucleus, the loss-of-function mutant UBLCP1 (peach) leads to an increase in proteasome assembly. A higher number of proteasomes are translocated to the cytosol, where they become active. This is reflected in increased protein degradation. The TF released from the ER is degraded, which leads to a decrease in the number of TF molecules entering the nucleus and a decrease in transcription levels of proteasome subunits and of *UBLCP1*.

In yeast and plants, the coordinated expression of proteasome subunit genes is controlled by unrelated but functionally analogous transcription factors, Rpn4 [39, 40] and NAC53/78 [41], respectively. In mammals, there are different analogous transcription factors. NRF1 (Nuclear Factor Erythroid-derived 2-Related Factor 1) is a central regulator of proteasome expression in the presence of proteasomal dysfunction [39, 40]. Under normal conditions, NRF1 is a type II integral ER membrane protein continuously retro-translocated from the ER back to the cytosol via the ER-associated protein degradation (ERAD) pathway, where it is rapidly ubiquitinated and degraded by the proteasome [39, 40, 42]. With proteotoxic stress, NRF1 is proteolytically released from the ER, enters the nucleus, and binds to antioxidant response element (ARE) core sequences (5’-TGA[C/T]NNNGC-3’) [43, 44] found upstream of most proteasome subunit genes. In this study, overactivation of the proteasome induced by mutant UBLCP1 is countered by downregulation of different subunits of the proteasome and UBLCP1. Here, it was determined that ARE sequences exist in the promotor of all subunits genes investigated as previously described in the literature [45] and also within the promotor of *UBLCP1* (5’-TGACACAGC-3’ at ~360 bp from the start site). Overactivation of the proteasome in cells due to defective de-phosphorylation by mutant UBLCP1 leads to degradation of a common transcription factor in the cytosol, thus leading to a coordinated downregulation of levels of core proteasome subunits and associated regulators, such as UBLCP1, as a protective mechanism (Fig. 6). This was demonstrated by the upregulation of these genes observed upon inhibition of the proteasome in proband cells. The significant increase in *UBLCP1* mRNA levels observed in treated control cells attests to the fact that *UBLCP1* expression is regulated by proteasomal activity. This, however, did not translate to a statistically significant result in proband cells. A potential reason for this observation could be nonsense-mediated decay of the truncated UBLCP1 transcript. This truncation could be leading to a reduced half-life of the transcript, which masks the increase in gene expression after treatment in proband cells. Western blot analysis revealed an increase in NRF1 protein levels after treatment with MG132, as previously reported by Redhakrishnan et al. in 2010 (see Supplementary Fig. 1).

While inhibition of the proteasome is widely investigated, very few studies report overactivation of proteasome function, and little is known about the ensuing impact on cells. During the acute phase of intracerebral hemorrhage, overactivation of the proteasome causes early degradation of the endoplasmic reticulum (ER) chaperone GRP78 and IκB proteins, which in turn activate NF-κB increasing neuroinflammation via expression of pro-inflammatory cytokines. According to Liew et al., overactivation of proteasome activity exacerbates ER stress [46]. A number of studies suggest an important role of ER stress in the pathophysiology of autism [47, 48]. The expression of ER stress-related genes *ATF4*, *ATF6*, *PERK*, *XBP1*, *CHOP*, and *IRE1* is significantly increased in the middle frontal gyrus of ASD subjects [49]. Fujita et al. [50] state that low levels of ER stress might alter membrane trafficking of synaptic functional molecules possibly leading to ASD pathophysiology. A missense mutation in *SLC6A1*, encoding GABA transporter 1 (GAT-1) in a patient with epilepsy and ASD was recently described with resultant relocation of the mutated protein to the ER. The increased ER retention, along with excessive ER-associated degradation, led to reduced GABA uptake function in this case [51].

Emerging evidence suggests that ASD pathophysiology may be linked to dysfunction of GABAergic inhibitory signaling in the brain. One of the GABAA receptor subunits, GABAAα1, exhibits a significant reduction at the protein level in the frontal cortex of ASD subjects [52]. Using postmortem brain samples and mouse primary cortical neurons, Crider et al. demonstrate a critical role for UPS-mediated mechanisms in the post-translational regulation of GABAAα1. Conversely, expression of the E3 ligase SYVN1 associated with GABAAα1 was higher in tissue samples from ASD compared to controls [49]. Overall, excessive UPS-mediated GABAAα1 turnover in ASD might lead to dysfunction of GABA signaling in cortical neurons.

These findings, while promising, remain distanced from the context of ASD due to tissue specificity differences between human fibroblasts and neurons. Nonetheless, it establishes the framework for future work in which iPSCs would be generated from control and proband fibroblasts prior to differentiation to neuronal and glial cellular subtypes. Only then can one assess the ensuing effects on neuronal and glial pathways. Emerging evidence suggests that proteasomes are relatively enriched in the brain and may play critical roles in neuronal physiology [53, 54]. A defective regulator such as ubiquitously expressed UBLCP1 may impact various aspects of neuronal development, including neurogenesis, differentiation, and synapse formation. It could also affect numerous post-developmental functions, such as synaptic plasticity and signaling. Other limitations of the current study include the use of male subjects only. This hinders the assessment of sex-specific differences in proteasome activity, which have been previously reported [55, 56].

This is the first discovery of a mutation leading to overactivation of the proteasome in ASD. Overall, the present work suggests that under or overactivation of proteostasis may cause ASD. Compensatory changes necessary to maintain a viable rate of proteolysis probably contribute to the neurological symptoms of ASD. Neutralizing the effect of this mutation by using gentamicin to promote read-through of the premature stop codon in *UBLCP1* mRNA corrected the deficient phenotype in cells. This result is a prelude to using the less toxic drug, Ataluren, a small non-aminoglycoside molecule, as a safe and effective treatment for this patient, and perhaps additional cases of autism with premature stop codons in other genes.

### Supplementary information

Supplementary Figure 1 legend

Supplementary figure 1

## Data Availability

Raw whole exome sequencing data from this study are available through the NCBI Sequence Read Archive (BioProject accession number: PRJNA1047123). VCFs are available upon request from Dr. Maria Chahrour (maria.chahrour@utsouthwestern.edu).
