# Supplementary material for: A novel autism-associated UBLCP1 mutation impacts proteasome regulation/activity
Source: Transl Psychiatry. 2023 Dec 21;13:404. doi: 10.1038/s41398-023-02702-0 (PMC10739866; doi:10.1038/s41398-023-02702-0)
Supplement: Supplementary file 1 — Supplementary Figure 1 legend [file 41398_2023_2702_MOESM1_ESM.docx]

**SUPPLEMENTARY LEGEND**

**Supplementary figure 1.** Increase in cleaved NRF1 (60 kDa) levels detected by Western blot in fibroblasts from proband treated with 1µM MG132 for 10h compared to fibroblasts from proband only treated with DMSO. The cleaved NRF1 form (60kDa) is the active form of the transcription factor. NRF1 and Vinculin (loading control) were detected by Western blot (n=1 each; Western blot repeated twice as seen in left panel). Equivalent amounts of total protein were loaded into each lane. Quantification is shown in the bar graph on the right.
