# Supplementary figures and images for: A novel autism-associated UBLCP1 mutation impacts proteasome regulation/activity
Source: Transl Psychiatry. 2023 Dec 21;13:404. doi: 10.1038/s41398-023-02702-0 (PMC10739866; doi:10.1038/s41398-023-02702-0)

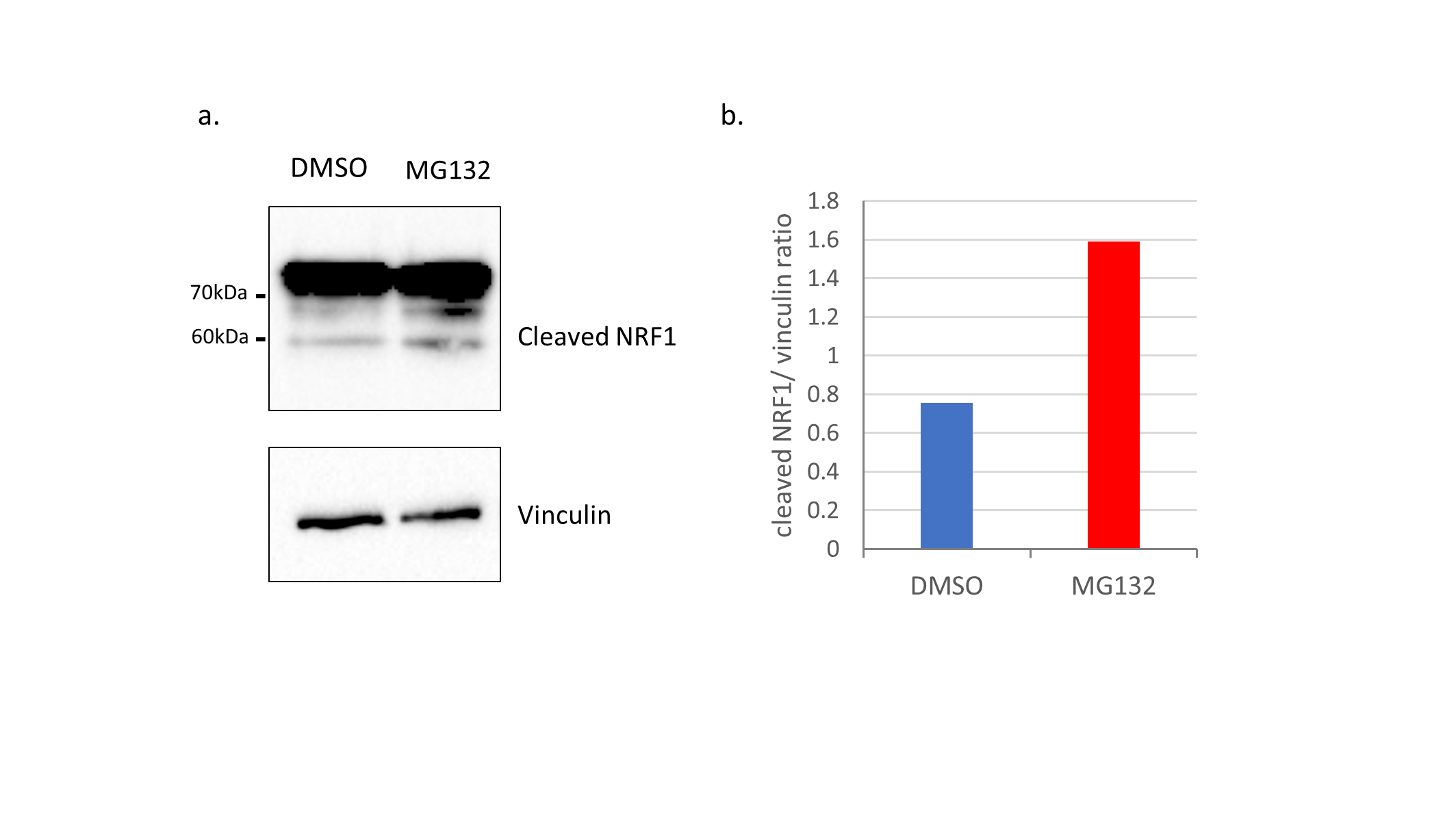

Supplement: Supplementary file 2 — Supplementary figure 1 [file 41398_2023_2702_MOESM2_ESM.tif]
